# Prediction of loss of heterozygosity in oral cavity dysplasia through vascular pattern

**DOI:** 10.3389/froh.2026.1829880

**Published:** 2026-07-09

**Authors:** Francesco Carlo Tartaglia, Giovanna Rossi, Luca Mainardi, Haiyang Wang, Carlo Resteghini, Funda Goker, Luigi Lorini, Cristina Gurizzan, Alberto Paderno, Paolo Bossi

**Affiliations:** 1 Department of Maxillofacial Surgery, University Hospital of Parma, Parma, Italy; 2 Department of Biomedical, Surgical and Dental Sciences, University of Milan, Milan, Italy; 3 Department of Electronics, Information and Bioengineering, Politecnico di Milano, Milan, Italy; 4 Department of Biomedical Sciences, Humanitas University, Milan, Italy; 5 Istituto di Ricovero e Cura a Carattere Scientifico (IRCCS) Humanitas Research Hospital, Rozzano, Milan, Italy; 6 UOC Maxillo-Facial Surgery and Dentistry, Fondazione IRCCS Ca' Granda, Ospedale Maggiore Policlinico, Milan, Italy; 7 Department of Oral and Maxillofacial Surgery, Faculty of Dentistry, Istanbul Aydın University, Istanbul, Turkey; 8 Otorhinolaryngology Unit, IRCCS Humanitas Research Hospital, Milan, Italy

**Keywords:** AI-Assisted imaging, artificial intelligence in oral oncology, loss of heterozygosity (LOH), machine learning, optical imaging (OI), oral dysplasia, vascular biomarkers, vascular pattern

## Abstract

**Background and purpose:**

Loss of Heterozygosity (LOH) is a key genetic alteration associated with progression of oral cavity dysplasia to oral squamous cell carcinoma, yet non-invasive methods to predict LOH status are lacking. The aim of this study is to investigate whether vascular pattern abnormalities detected through Narrow Band Imaging (NBI) can predict LOH status in oral dysplasia using machine learning-based quantitative analysis.

**Methods:**

This was a retrospective analysis of prospectively collected data from the IMPEDE multicenter clinical trial (NCT04504552). The study sample included 31 patients with oral potentially malignant disorders (13 LOH-positive, 18 LOH-negative) and 125 region-of-interest (ROI) images. Predictor variables were quantitative vascular morphology features: vessel length, number of intersections, tortuosity, branching angles, fractal dimensions, which were extracted from NBI images using the Jerman Vesselness Filter and pvbm library. Main outcome variable was LOH status (positive *versus* negative) by genetic testing per EPOC trial criteria. Patient demographics, smoking status, alcohol consumption were the covariates. A Support Vector Machine (SVM) classifier with class balancing and probability calibration was trained using Leave-One-Group-Out Cross-Validation (LOGO-CV) at the patient level.

**Results:**

Of 31 patients (median age 65 years; 19 males, 12 females), 13 (42%) were LOH-positive. Significant differences in vascular morphology were observed between LOH-positive and LOH-negative samples. The SVM classifier achieved a patient-level accuracy of 77.4% (24/31 correctly classified), with an area under the ROC curve (AUC) of 0.75.

**Conclusions:**

Non-invasive quantitative vascular pattern analysis from NBI images demonstrates good discriminative ability for predicting LOH status in oral dysplasia. This approach has potential as an adjunct diagnostic tool for early cancer risk stratification, potentially reducing the need for invasive biopsies.

## Introduction

Oral Potentially Malignant Disorders (OPMDs) are categorized into various subtypes, including leukoplakia and erythroplakia (1–3). These lesions carry a risk of progressing to oral squamous cell carcinoma (OSCC), although many cases show benign hyperkeratosis. Overall, reports of progression to cancer vary widely from 6% to 36% at an annual rate of 1.36% (95% CI, 0.69%–2.03%) (4). Epithelial dysplasia, marked by architectural and cytological abnormalities, significantly increases malignancy risk (5). The severity of dysplasia is graded as mild, moderate, or severe, with higher grades associated with greater risk of progression to cancer. Oral cavity dysplasia represents a significant concern in oral pathology due to its potential to develop into OSCC, which poses a considerable healthcare burden worldwide (1, 6).

Oral epithelia dysplasia affects an estimated 2.5% to 5% of the population, with higher risk groups potentially reaching 10% (7, 8) while males are more frequently affected than females (9, 10). Furthermore, incidence is higher in older adults, particularly those over 40 years of age (11) and in regions with high tobacco (e.g., Australia, Eastern and Western Europe) and betel nut (e.g., South and East Asia) consumption (9). Genetic, environmental, and lifestyle factors influence the transition from dysplasia to malignancy. Notable risk factors include tobacco use, alcohol consumption, and human papillomavirus (HPV) infection (12, 13).

However, clinical appearance alone is an unreliable predictor of malignancy (1, 5), and histological grading remains subjective with limited predictive value (14). The 2017 World Health Organization (WHO) classification for oral epithelial dysplasia (OED) is complex and lacks standardized grading criteria, leading to variability in diagnosis (15).

Additionally, conventional biopsies suffer from sampling bias, as they may not capture the full extent of dysplastic lesions (16–18). Given these challenges, molecular markers and advanced imaging technologies are essential to enhance diagnostic accuracy.

Understanding the mechanisms underlying dysplasia, particularly genetic alterations such as loss of heterozygosity (LOH) in candidate chromosome-arm regions, is crucial for enhancing early diagnosis and treatment strategies. Genetic alterations, particularly LOH at critical chromosomal loci, have been identified as significant indicators of malignancy risk. LOH, the loss of one allele in a gene pair, often leads to the inactivation of tumor suppressor genes (19). Chromosomal regions such as 3p, 4q, 9p, 17p, and 18q frequently exhibit LOH in oral cancers (20, 21). In particular, LOH at 3p and 9p, which involves tumor suppressor genes such as TP53, CDKN2A and PTEN, has been linked to increased cancer risk (13, 14, 22, 23). Research suggests that LOH results from large chromosomal alterations rather than point mutations, playing a role in tumor immune evasion (24). Although LOH itself is not a direct therapeutic target, immune pathways affected by LOH may offer pharmacological intervention opportunities to mitigate cancer risk (23).

LOH also impacts the microvascular environment of oral dysplasia, influencing vascular integrity and angiogenesis (3, 24). It deregulates angiogenic pathways, leading to abnormal neovascularization and alterations in intraepithelial papillary capillary loops (IPCLs) (5, 19, 21, 25). Notable vascular changes associated with LOH include increased angiogenesis, structural irregularities, and perivascular inflammation. The imbalance between pro-angiogenic and anti-angiogenic factors, leads to excessive and disorganized vascular proliferation (3, 25–27). As a consequence, the affected vessels become dilated, elongated, and irregular. In more advanced stages, such as severe dysplasia or carcinoma *in situ* (CIS), there is evidence of chaotic angiogenesis and loss of vascular integrity (25–27). Lastly, genetic instability from LOH attracts inflammatory cells, altering the vascular microenvironment further contributing to the pathological process (25).

Narrow Band Imaging (NBI) has emerged as an advanced diagnostic tool for detecting vascular changes associated with LOH, offering improved accuracy in identifying high-risk lesions (1, 6, 26, 28). NBI is a non-invasive optical diagnostic technology that enhances mucosal and submucosal vascular visualization by utilizing blue (415 nm) and green (540 nm) narrow-band light. This technology highlights blood vessel contrast, aiding in the detection of neoplastic changes (28). NBI provides real-time imaging without requiring contrast agents, making it a convenient and stress-free option for routine screenings.

According to the classification by Tirelli et al. (29), six vascular patterns have been identified using NBI, corresponding to different histological and anatomical characteristics of the oral mucosa. It has been noted that the neoplastic transformation results in the complete destruction of vascular structures, with remnants of IPCLs appearing as dark green spots or dilated, winding vessels. In ulcerated cancerous lesions, necrotic regions often obscure vascular structures, though tumor peripheries may still display IPCL remnants.

Given the limitations of subjective histopathological grading, this study aims to explore the correlation between LOH and vascular pattern abnormalities using machine learning techniques for enhanced diagnostic accuracy. By analyzing IPCLs and vascular patterns in oral dysplasia, this research seeks to bridge the gap between advanced imaging and molecular pathology, offering clinicians an objective and reproducible diagnostic approach.

## Materials and methods

### Dataset description

The dataset used in this study consists of 125 region-of-interest (ROI) images obtained from 31 patients. They were collected only in selected Centers (Spedali Civili Brescia, Humanitas Hospital Milan) as part of the IMPEDE clinical trial (NCT04504552), a prospective multicenter study investigating an immunopreventive strategy with avelumab given intravenously for 4 cycles for oral potentially malignant disorders (OPMDs) characterized by pre-specified high risk LOH positivity (30). All images were annotated and pre-processed for vascular pattern extraction. The images were labeled according to the patient's LOH status, with 13 patients identified as LOH positive and 18 as LOH negative [LOH was defined by PCR-based microsatellite analysis at loci 3p14, 9p21, 4q, 8p, 11q, 13q, and 17p per the EPOC trial protocol (31)]. Inclusion in the trial required histologically confirmed dysplasia on incisional biopsy; LOH-negative patients served as the internal comparison cohort (30).

The median age was 65 years, with an age range of 40 to 82 years. The gender distribution consisted of 19 males (61.3%) and 12 females (38.7%). Regarding smoking status, 21 patients (67.7%) were non-smokers, 6 (19.35%) were former smokers, and 4 (12.9%) were current smokers. Among the current smokers, pack-year data ranged from 15 to 90, with one patient reporting a notably high value of 90 pack-years. For alcohol status, 24 patients (77.42%) were non-drinkers and 7 (22.58%) were current drinkers (Table 1).

**Table 1:** LOH-stratified summary by gender for smoking and alcohol-related variables. Values represent count within each LOH status group.

Characteristic	Male (*n* = 19)	Female (*n* = 12)	Total
Smoking Status: Current Smoker	LOH = 0: 2 LOH = 1: 0	LOH = 0: 2 LOH = 1: 0	LOH = 0: 4 LOH = 1: 0
Smoking Status: Former Smoker	LOH = 0: 4 LOH = 1: 1	LOH = 0: 0 LOH = 1: 1	LOH = 0: 4 LOH = 1: 2
Smoking Status: Non-Smoker	LOH = 0: 3 LOH = 1: 9	LOH = 0: 7 LOH = 1: 2	LOH = 0: 10 LOH = 1: 11
Current Smoker (Binary): No	LOH = 0: 7 LOH = 1: 10	LOH = 0: 7 LOH = 1: 3	LOH = 0: 14 LOH = 1: 13
Current Smoker (Binary): Yes	LOH = 0: 2 LOH = 1: 0	LOH = 0: 2 LOH = 1: 0	LOH = 0: 4 LOH = 1: 0
Alcohol Status: Current Drinker	LOH = 0: 4 LOH = 1: 0	LOH = 0: 2 LOH = 1: 1	LOH = 0: 6 LOH = 1: 1
Alcohol Status: Non-Drinker	LOH = 0: 5 LOH = 1: 10	LOH = 0: 7 LOH = 1: 2	LOH = 0: 12 LOH = 1: 12

LOH, Loss of Heterozygosity; LOH = 0, LOH-negative; LOH = 1, LOH-positive.

### ROI extraction

A Python script was developed to delineate Regions of Interest (ROIs) on the NBI dataset using polygonal boundaries. The ROIs were initially drawn and subsequently reviewed by two otolaryngologists (one with < 5 years and one with > 10 years of experience in endoscopic diagnosis with NBI). A single ROI was defined for each 2D image. Each ROI, represented as a polygon, delineated the region for vessel-structure analysis, specifically encompassing the hyperkeratotic leukoplakia and the perilesional area where vessels are more clearly visible. The number of ROIs per patient varied due to the fact that there are limited points of view (POVs) from which the lesion can be effectively visualized. Although image transformations such as rotations or flips could be applied, these would not generate new, independent ROIs, as the underlying vascular pattern remains unchanged. For all computations, a binary mask was generated from the polygon and analysis was strictly restricted to the interior of the ROI (i.e., only pixels within the polygon were included; any signal outside the polygon was excluded). Vessel-structure metrics were then computed separately for each ROI, treating each ROI as an independent unit.

### Image preprocessing and vessel analysis

To enhance the visibility of vascular structures within the ROIs, a standardized image pre-processing pipeline was implemented. Initially, all images were converted to grayscale to reduce computational complexity and focus on intensity-based features. Local contrast was then improved using Contrast Limited Adaptive Histogram Equalization (CLAHE), which adaptively redistributes pixel intensities within small, context-specific tiles to enhance fine details while preventing over-amplification of noise (32). To further improve edge/vessel definition, an unsharp masking filter (*α* = 1) was applied. Finally, noise reduction was achieved through a median filter employing a disk-shaped structuring element with a radius of 3 pixels, which preserves edge integrity while attenuating high-frequency artifacts.

On the preprocessed images, the Jerman Vesselness Filter was employed to enhance vascular structures in 2D images and to facilitate the visualization of tubular formations. Originally developed for 3D medical imaging applications (33), the filter has been adapted for use with 2D modalities, such as histological or microscopic images. This adaptation includes modifications to the Hessian matrix computations and eigenvalue normalization to optimize the detection of 2D vessel-like structures.

**Figure 1:**
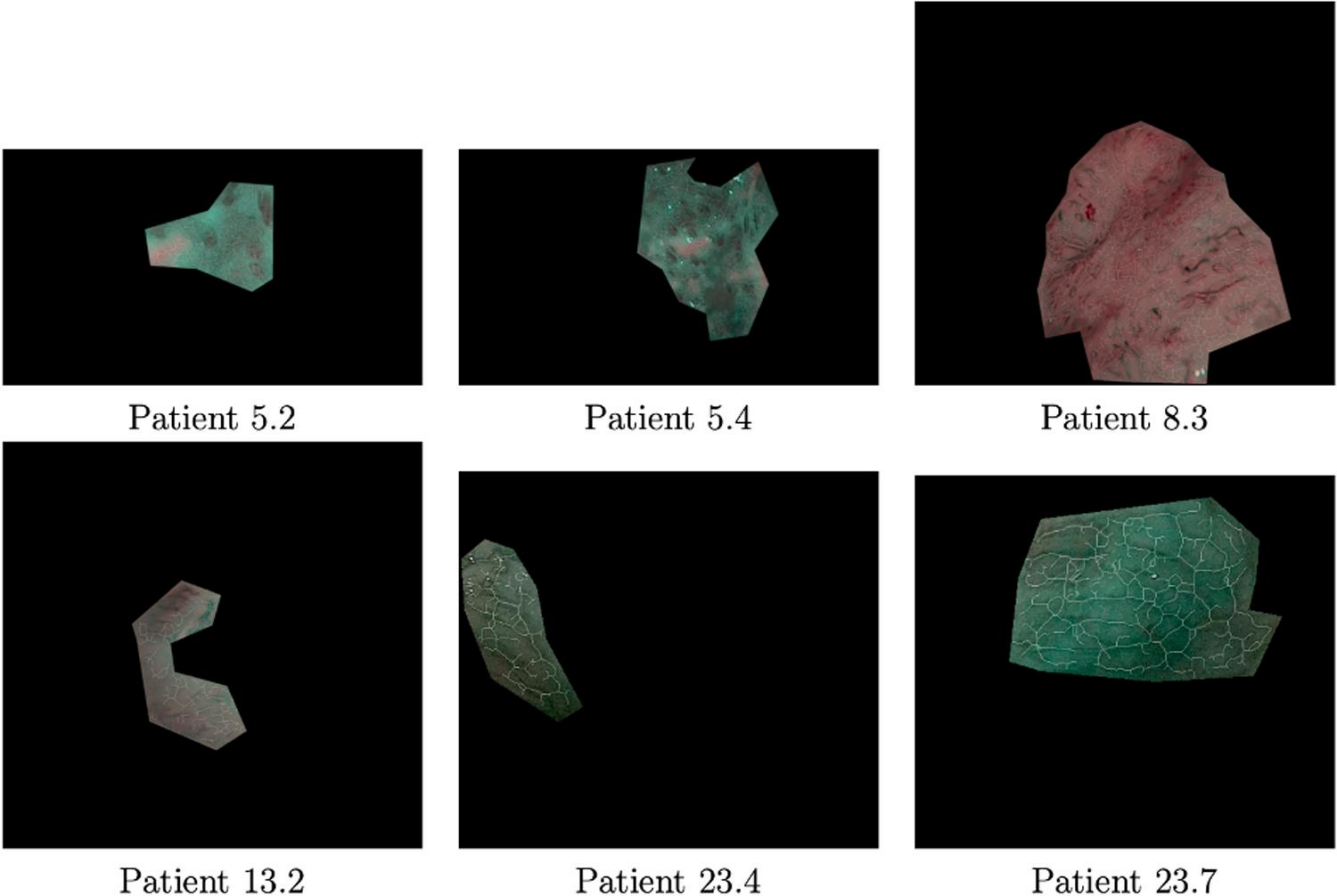
Superimposed skeletonized vessels on the original images for selected patients. These images highlight the intricate details of the vascular structures, including fine capillaries and their complex networks.

Following enhancement, the output of the Jerman filter was subjected to skeletonization to extract the centerlines of the vascular patterns. Representative vascular maps obtained by the described approach, are overlaid on the original fibroscopic images in Figure 1.

### Feature extraction

The vascular structures were analyzed using geometric and fractal-based methods via the pvbm library (version 2.9.9.5) (34, 35). Key extracted features included:

- Number of endpoints and intersections.
- Vessel length, perimeter, and tortuosity.
- Branching angles and their variability.
- Fractal dimensions (D0, D1, D2) and spectrum length (SL).

To address missing values in extracted vascular features, particularly for D0, D1, D2, and SL, the Multiple Imputation by Chained Equations (MICE) method was employed (36).

These missing values primarily arose from occasional failures in the feature extraction algorithms, which were unable to compute specific descriptors for certain samples. MICE provides a robust imputation approach by creating multiple plausible values for missing data based on existing patterns in the dataset. This method improves data integrity, prevents information loss, and ensures that statistical analyses and machine learning models are trained on a more complete dataset. By applying MICE, we mitigated potential biases and improved the reliability of the vascular pattern analysis in distinguishing LOH-positive from LOH-negative cases.

### Statistical analysis and classification

Normality testing was performed using the Shapiro–Wilk test to assess the distribution of vascular features within LOH-positive and LOH-negative groups. Based on normality, appropriate statistical tests (independent t-tests or Mann–Whitney U tests) were applied to identify characteristics that differed significantly between the two groups.

A Support Vector Machine (SVM) classifier with class balancing and Platt probability calibration was trained to predict LOH status (positive vs. negative) from standardised image-level vascular features. Hyperparameters were optimised via GridSearchCV with 5-fold cross-validation and F1 score as the selection metric; the search space included kernel type (linear, RBF, polynomial), regularisation parameter C (0.01–1,000), and kernel coefficient gamma (scale, auto). Platt scaling fitted a sigmoid to the SVM decision scores via internal 5-fold cross-validation on training data. To prevent data leakage, feature selection was performed within each Leave-One-Group-Out Cross-Validation (LOGO) fold exclusively on the training subset, ensuring the held-out patient's data had no influence on feature selection at any stage.

Model evaluation employed a LOGO approach at the patient level, ensuring that all images from the same patient appeared only in either the training or testing set. For each held-out group, significant features were re-selected and the model was retrained from scratch. The final classification per patient was derived by averaging the predicted probabilities from all their associated images; if the mean exceeded 0.5, the patient was classified as LOH-positive, otherwise LOH-negative.

Model performance was assessed both at the image and patient levels. Standard classification metrics, including accuracy, precision, recall, F1 score, and confusion matrix, were computed on patient-level predictions. Additionally, all trained models and result outputs were saved for reproducibility and further analysis.

### Model evaluation and performance

To evaluate the performance of the classification model, standard evaluation metrics were employed. These included the Receiver Operating Characteristic (ROC) curve, the Area Under the Curve (AUC), and the confusion matrix. The ROC curve and AUC were used to quantify the model's ability to distinguish between LOH-positive and LOH-negative samples across varying decision thresholds. The confusion matrix provided a detailed breakdown of true and false classifications.

## Results

### Feature analysis and statistical comparison

The extracted vascular features were compared between LOH-positive and LOH-negative groups. Normality was assessed using the Shapiro–Wilk test, which indicated that none of the tested features followed a normal distribution in either group (see Table 2).

**Table 2:** Statistical comparison of vascular features between LOH groups. Shapiro–Wilk tests were used to assess normality. Because none of the features followed a normal distribution in both groups, Mann–Whitney U tests were used to compare features between LOH-positive and LOH-negative groups.

Feature	Shapiro–Wilk *p*-value (LOH=0)	Shapiro–Wilk *p*-value (LOH=1)	Mann–Whitney U Statistic	*p*-value
n endpoints	*<*0*.*0001	*<*0*.*0001	2,362.5	**0**.**0356**
n intersections	*<*0*.*0001	*<*0*.*0001	2,378.0	**0**.**0294**
median tortuosity	0.0155	*<*0*.*0001	1,550.0	**0**.**0493**
length	*<*0*.*0001	*<*0*.*0001	2,452.0	**0**.**0109**
perimeter	*<*0*.*0001	*<*0*.*0001	2,427.0	**0**.**0155**
mean branching angle	*<*0*.*0001	*<*0*.*0001	2,005.0	0.7417
std branching angle	*<*0*.*0001	*<*0*.*0001	1,800.0	0.4955
median branching angle	*<*0*.*0001	*<*0*.*0001	2,025.0	0.6681
D0	0.0007	*<*0*.*0001	1,755.0	0.3657
D1	0.0002	*<*0*.*0001	1,647.0	0.1499
D2	0.0002	*<*0*.*0001	1,962.0	0.9073
SL	0.0002	*<*0*.*0001	1,777.0	0.4263

The bold values provided in the Table indicate the statistically significant differences (*p* < 0.05).

Accordingly, non-parametric Mann–Whitney U tests were used to evaluate differences between the groups.

Significant differences were found in several vascular features. LOH-negative samples showed significantly higher numbers of vascular endpoints and intersections (both *p* < 0.05), as well as greater vessel length and perimeter (*p* < 0.05), indicating a more extensive and organized vascular network. Additionally, LOH-positive samples exhibited significantly increased vessel tortuosity (*p* = 0.0493). These results suggest distinct vascular remodeling patterns associated with LOH status.

### Classification performance

**Figure 2:**
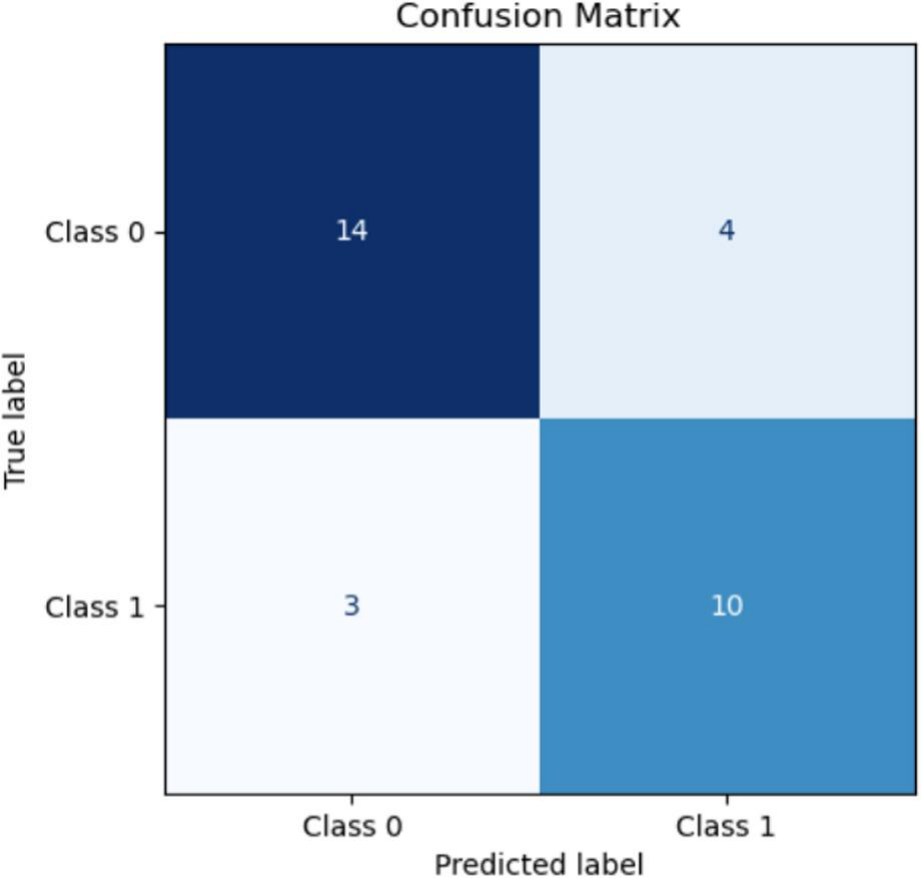
Confusion matrix of the SVM classifier. Class 0 = LOH negative, Class 1 = LOH positive.

Figure 2 illustrates the confusion matrix and overall classification performance of the SVM classifier. The model achieved an accuracy of 77.4%, with a sensitivity of 76.9% and a specificity of 82.5%, indicating reliable differentiation between LOH-positive and LOH-negative cases. For the LOH-positive class, precision was 71.4% and the F1-score was 0.741 (Table 3), confirming a balanced trade-off between precision and recall.

**Table 3:** Classification performance metrics.

Metric	Value
Accuracy	77.4%
Precision	71.4%
Recall (sensitivity)	76.9%
F1 score	74.1%

Given the clinical implications of misclassification, the model was explicitly optimized using the F1 score. This decision was driven by the need to prioritize the correct identification of LOH-positive patients. In a diagnostic context, it is preferable to classify a patient as LOH-positive (i.e., potentially at risk) and proceed with further testing, even if the result later proves negative, than to incorrectly classify an LOH-positive patient as negative and consequently miss the opportunity for early intervention and surveillance.

This strategy reflects a conservative and patient-centered approach, favoring sensitivity while maintaining an acceptable level of precision.

**Figure 3:**
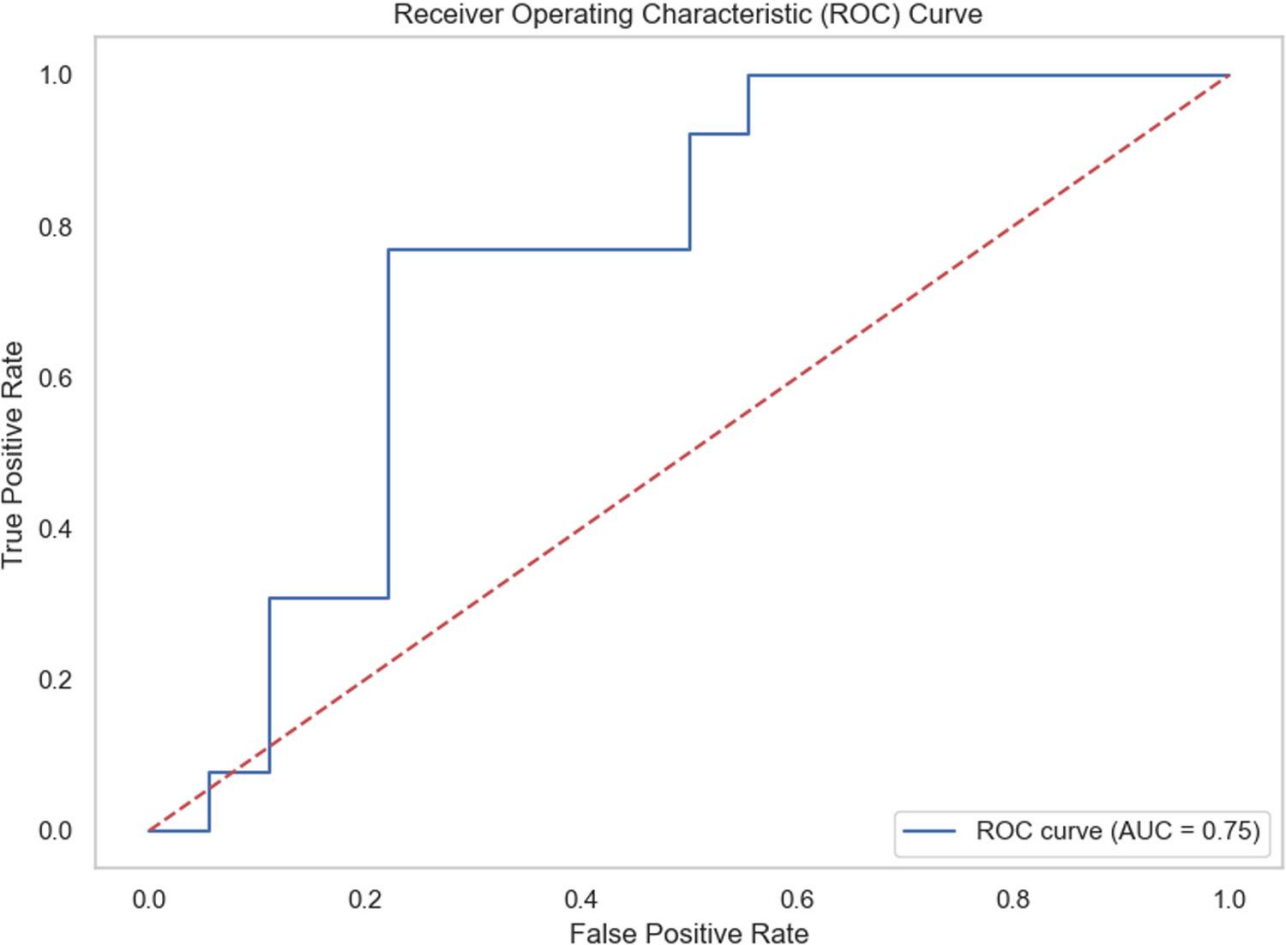
Receiver Operating Characteristic (ROC) curve for the SVM classifier.

Figure 3 presents the ROC curve. The model achieved an AUC of 0.75, demonstrating strong discriminative capability. The curve's steep initial rise reflects high sensitivity, which is essential for minimizing false negatives in a clinical setting. Additionally, the model learning curves, demonstrating stability and lack of overfitting, are provided in the Supplementary Material.

## Discussion

The findings of this study emphasize the importance of vascular pattern analysis in identifying loss of heterozygosity in oral cavity dysplasia. The significant differences observed in vascular features between LOH-positive and LOH-negative samples highlight the role of angiogenesis and microvascular alterations in malignant progression. The classification model, employing a SVM, demonstrated high accuracy, reinforcing the potential of machine learning techniques in enhancing early diagnosis and risk stratification.

One of the primary findings of this study was the marked disruption of organised vascular architecture in LOH-positive lesions, evidenced by significantly reduced endpoint counts, intersections, vessel length, and perimeter, alongside increased tortuosity (Table 2). These quantitative differences are consistent with LOH-driven angiogenic dysregulation: loss of tumour suppressor activity at 3p14 and 9p21 promotes aberrant VEGF signalling, fragmenting the orderly IPCL network and replacing it with dilated, tortuous, and irregularly shaped vessels, substituting the regular corkscrew morphology characteristic of normal epithelium. Notably, the features most discriminative for the SVM classifier were tortuosity, vessel length, endpoint count, and intersection density, which align closely with the vascular alterations expected on biological and clinical grounds, reinforcing the interpretability of the model and suggesting that the learned decision boundary reflects pathophysiological signal rather than spurious statistical associations. This pattern is further consistent with prior evidence linking aberrant IPCL morphology to malignant transformation (5, 21) and with the broader literature on microvascular instability as a consequence of LOH-associated genomic alterations (3, 25). Collectively, these vascular features represent biologically grounded, imaging-accessible surrogates of the underlying molecular tissue state, supporting their utility as non-invasive biomarkers for LOH-driven dysplastic progression.

From a clinical perspective, these vascular abnormalities could represent a non-invasive, early biomarker for dysplasia severity and malignant potential. In current clinical practice, the decision to biopsy a lesion is often based on visual inspection and subjective assessment, which are prone to variability and limited sensitivity (37, 38). The identification of objective vascular criteria could offer clinicians a reproducible and evidence-based tool to prioritize high-risk lesions for biopsy and early intervention. In particular, lesions with preserved vascular organization may be safely monitored, potentially reducing unnecessary biopsies and associated patient morbidity. Furthermore, the ability to stratify patients based on vascular alterations could significantly impact follow-up strategies. Patients with LOH-positive lesions and corresponding microvascular disruption may benefit from closer surveillance and adjunctive therapies, including chemoprevention or early surgical excision. Conversely, patients with LOH-negative lesions and normal vascular patterns could be managed more conservatively, optimizing resource allocation and patient care pathways.

Recent evidence supports the utility of vascular pattern assessment as a biomarker for dysplastic progression. Studies have shown that abnormal IPCLs, visualized through NBI, are strongly associated with high-grade dysplasia and early carcinoma (39, 40). Meta-analyses have confirmed that NBI, when assessing vascular abnormalities, achieves high sensitivity and specificity for detecting malignant transformation in OPMDs (41).

The statistical analyses offered strong evidence of differences in vascular patterns, which could be used as objective variables in the clinical diagnostic decision-making process. The Shapiro–Wilk test confirmed the non-normal distribution of the data, necessitating appropriate statistical adjustments. The Mann–Whitney U test identified key features such as vessel length, number of intersections, number of endpoints, and tortuosity as significantly different between LOH-positive and LOH-negative cases. These results suggest that microvascular alterations could serve as potential biomarkers for early identification of high-risk lesions, enabling targeted interventions and improved patient outcomes.

The clinical implications of this study are significant. While histopathological evaluation remains the gold standard for dysplasia assessment, the non-invasive nature of vascular pattern analysis provides an adjunctive tool for risk stratification. The ability to classify LOH-positive and LOH-negative lesions with high accuracy suggests that vascular biomarkers identified by NBI may be used for patient monitoring and early intervention. Integrating this approach into routine clinical workflows could improve lesion management and reduce unnecessary biopsies (15).

Differences in vascular characteristics between LOH-positive and LOH-negative samples highlight the significant role of microvascular alterations in malignant progression.

Specifically, the quantity of endpoints, intersections, median tortuosity, and vessel length are most strongly associated with LOH. Furthermore, NBI shows superior diagnostic accuracy to conventional white-light endoscopy for distinguishing benign from malignant lesions in the larynx and pharynx, and enables visualization of subtle subepithelial vascular patterns that are otherwise difficult to appreciate (42, 43). Especially, when in combination with contact endoscopy, a higher accuracy has been shown in distinguishing benign vs. malignant lesions of the larynx and pharynx. In parallel, the emerging field of videomics proposes deep-learning pipelines that operate on endoscopic video to detect, segment, and characterize lesions as outlined by Paderno et al., pointing toward human–computer collaboration at the scope level of vascular features highlighted by NBI (44, 45). Another important consequence of this link between vascular patterns and LOH could be the possibility of targeting these vascular abnormalities with targeted agents. However, even if the altered angiogenetic pathways have been demonstrated in OPMD and the proangiogenic factors as VEGF and angiopoietin-2 are highly expressed in oral cavity cancers, till now no antiangiogenic strategy has been tested with preventative aims (46).

Despite the promising results, the study presents several limitations. First, the dataset size is relatively small, with only 125 regions of interest (ROIs) analyzed from 31 patients as reported in Table 1. This limitation affects the generalizability of the model and necessitates further validation on larger cohorts. This study should therefore be interpreted as a pilot and hypothesis generating investigation for potential prospective clinical trials. In the context of oral cancer diagnostics specifically, several comparable NBI-based and vascular feature studies have similarly been constrained by limited patient numbers, reflecting the inherent difficulty of prospective tissue collection with paired molecular annotation (25, 27).

Additionally, the statistical feature selection (Mann–Whitney U and t-tests) was applied at the image level, where multiple ROIs from the same patient share a single LOH label, technically violating the independence assumption. This is an acknowledged limitation of the exploratory filter applied within training folds only and does not affect the primary patient-level ML evaluation. Future studies should incorporate multi-institutional datasets to improve robustness and reliability. Additionally, while NBI effectively enhances vascular visualization, hyperkeratotic lesions may obscure underlying microvascular structures, potentially impacting the accuracy of vascular feature extraction. The incorporation of complementary imaging modalities, such as Optical Coherence Tomography (OCT) or Confocal Laser Endomicroscopy (CLE), may improve diagnostic accuracy (28).

Another consideration is the subjectivity associated with manual annotation of vascular structures. Although the annotations were verified by specialists, interobserver variability remains a challenge in medical image analysis. The integration of automated vessel segmentation algorithms, incorporating deep learning techniques, could enhance reproducibility and standardization in vascular feature extraction (47). Future research should focus on developing robust, fully automated pipelines for real-time clinical application.

## Conclusion

This study highlights the potential of vascular pattern analysis as a non-invasive method for identifying LOH in oral cavity dysplasia. By leveraging machine learning techniques and advanced imaging modalities, significant differences in vascular complexity, connectivity, and morphology were identified between LOH-positive and LOH-negative samples. The classification model achieved high accuracy, demonstrating the feasibility of using vascular biomarkers for early dysplasia assessment. The identification of microvascular abnormalities associated with LOH may provide clinicians with a valuable tool to direct the clinical choices, guiding biopsy decisions, prioritizing high-risk lesions for early intervention, and enabling closer surveillance of dysplastic areas with disrupted vascular architecture. Conversely, lesions showing preserved vascular organization may be managed more conservatively, reducing unnecessary biopsies and patient morbidity.

While promising, these findings necessitate further research to enhance clinical applicability. Expanding dataset size, integrating multimodal imaging approaches, and incorporating automated segmentation techniques will be crucial in refining the predictive accuracy of vascular biomarkers. Additionally, validating these findings across larger, multi-institutional cohorts will strengthen their reliability and applicability in clinical settings.

Ultimately, vascular pattern analysis could become a powerful adjunct to histopathological evaluation, contributing to more personalized, timely, and effective management of patients with oral potentially malignant disorders, and improving long-term outcomes in oral cancer prevention.

## Data Availability

The data analyzed in this study is subject to the following licenses/restrictions: The data and code used in this study are available upon reasonable request. Requests to access these datasets should be directed to Francesco Carlo Tartaglia, francesco.tartaglia@st.hunimed.eu.
