# Supplementary material for: Prediction of loss of heterozygosity in oral cavity dysplasia through vascular pattern
Source: Front Oral Health. 2026 Jul 9;7:1829880. doi: 10.3389/froh.2026.1829880 (PMC13391842; doi:10.3389/froh.2026.1829880)

Supplementary Material

1 Supplementary Information

Model Learning Curve

To assess the model's generalization ability, a learning curve was plotted to evaluate performance across different training set sizes. Figure S1 shows the learning curve, which highlights the relationship between training accuracy and validation accuracy. The convergence of the two curves suggests that the model is not overfitting and has learned a stable representation of the vascular features. This curve can be considered acceptable considering the low number of available images.

Overall, the results indicate that vascular feature analysis, in combination with machine learning, provides an effective framework for LOH prediction. The strong AUC value, high classification accuracy, and well-behaved learning curve underscore the robustness of this approach in early cancer risk assessment.

2 Supplementary Figures

Supplementary Figure 1. Learning curve of the SVM classifier. The convergence of training and validation accuracy suggests effective generalization.

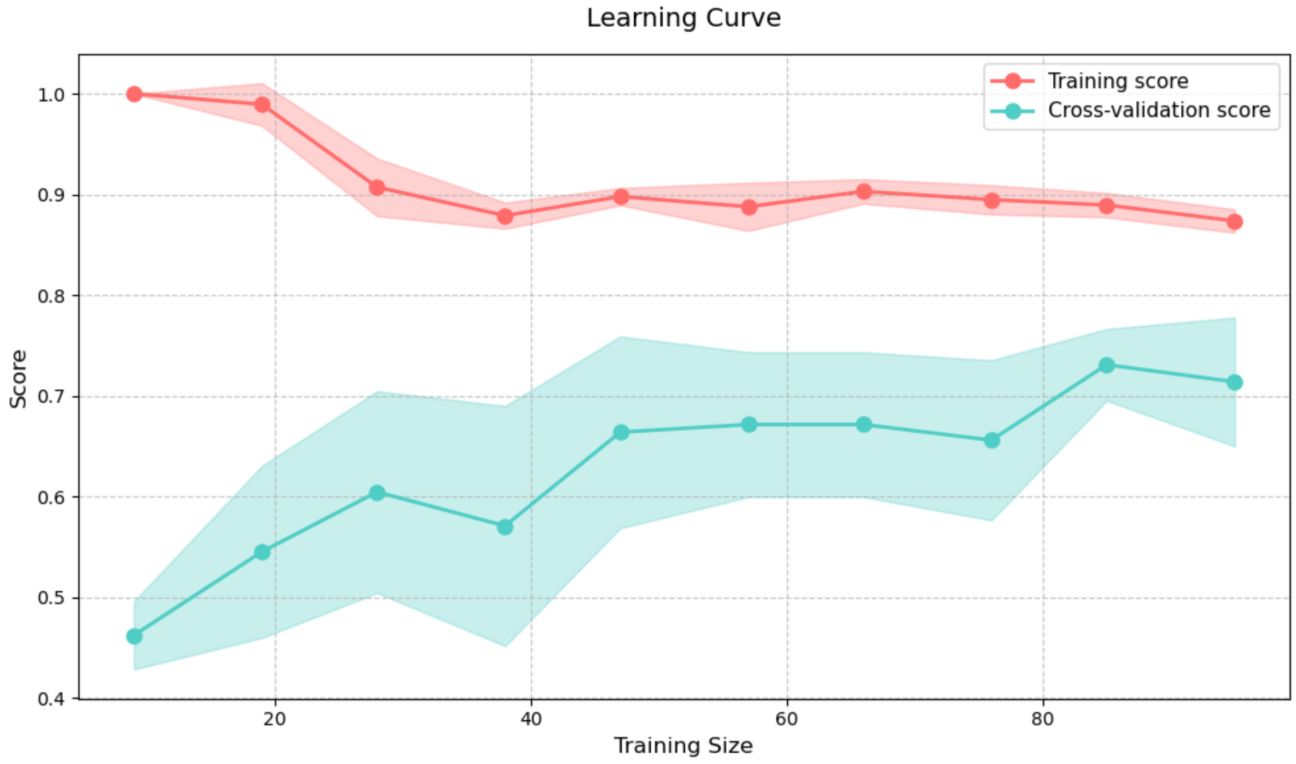

Supplement: Supplementary file 1 [file Datasheet1.pdf]
